# The effects of increased dose of hepatitis B vaccine on mother-to-child transmission and immune response for infants born to mothers with chronic hepatitis B infection: a prospective, multicenter, large-sample cohort study

**DOI:** 10.1186/s12916-021-02025-1

**Published:** 2021-07-13

**Authors:** Xiaohui Zhang, Huaibin Zou, Yu Chen, Hua Zhang, Ruihua Tian, Jun Meng, Yunxia Zhu, Huimin Guo, Erhei Dai, Baoshen Zhu, Zhongsheng Liu, Yanxia Jin, Yujie Li, Liping Feng, Hui Zhuang, Calvin Q. Pan, Jie Li, Zhongping Duan

**Affiliations:** 1 Artificial Liver Treatment Center, Beijing Youan Hospital, Capital Medical University, Beijing, China; 2 Beijing Municipal Key Laboratory of Liver Failure and Artificial Liver Treatment Research, Beijing, China; 3 Department of Obstetrics and Gynecology, Beijing Youan Hospital, Capital Medical University, Beijing, China; 4 Department of Liver Diseases, The Fifth Hospital of Shijiazhuang, Shijiazhuang, China; 5 Tongliao Infective Disease Hospital, Tongliao, China; 6 Department of Obstetrics and Gynecology, Taiyuan No. 3 Hospital, Taiyuan, China; 7 Department of Microbiology and Center of Infectious Disease, School of Basic Medical Sciences, Peking University Health Science Center, Beijing, China; 8 Division of Gastroenterology and Hepatology, Department of Medicine, New York University, Langone Health, NYU Grossman School of Medicine, New York, USA

**Keywords:** Hepatitis B vaccine, High dose, Mother-to-child transmission, Immune response

## Abstract

**Background:**

Appropriate passive-active immunoprophylaxis effectively reduces mother-to-child transmission (MTCT) of hepatitis B virus (HBV), but the immunoprophylaxis failure was still more than 5% under the current strategy. The study objective was to investigate the effects of high dose of HB vaccine on MTCT and immune response for infants born to hepatitis B surface antigen (HBsAg)-positive mothers.

**Methods:**

This was a prospective, multicenter, large-sample cohort study in four sites of China, and 955 pairs of HBsAg-positive mothers and their infants were enrolled in our investigation. The infants were given 10 μg or 20 μg HB vaccine (at age 0, 1, and 6 months) plus HB immunoglobulin (at age 0 and 1 month). Serum HBsAg, antibody to HBsAg (anti-HBs), and/or HBV DNA levels in the infants were determined at age 12 months. The safety of 20 μg HB vaccine was evaluated by adverse events and observing the growth indexes of infants.

**Results:**

Thirteen of 955 infants were HBsAg-positive at 12 months. Stratification analysis showed that immunoprophylaxis failure rates in the 20 μg group were not significantly different from the 10 μg group, whatever maternal HBV load was high or not. But the high dose of HB vaccine significantly reduced low-response rate (anti-HBs 10–100 IU/L) (*P* = 0.002) and middle-response rate (anti-HBs 100–1000 IU/L) (*P* = 0.022) and improved high-response rate (anti-HBs ≥ 1000 IU/L) (*P* < 0.0001) in infants born to mothers with HBV DNA < 5 log_10_ IU/mL. For infants born to mothers with HBV DNA ≥ 5 log_10_ IU/mL, 20 μg HB vaccine did not present these above response advantages. The 20 μg HB vaccine showed good safety for infants.

**Conclusions:**

The 20 μg HB vaccine did not further reduce immunoprophylaxis failure of infants from HBsAg-positive mothers, but increased the high-response and decreased low-response rates for infants born to mothers with HBV DNA < 5 log_10_ IU/mL.

**Trial registration:**

Chinese Clinical Trial Registry, ChiCTR-PRC-09000459

**Supplementary Information:**

The online version contains supplementary material available at 10.1186/s12916-021-02025-1.

## Background

Chronic hepatitis B virus (HBV) infection remains a serious threat to public health and is associated with cirrhosis and hepatocellular carcinoma (HCC) in China. It is estimated that the prevalence of hepatitis B surface antigen (HBsAg) in China is 5–6% at present, and about 70 million persons have chronic HBV infection, including 20–30 million chronic HB (CHB) patients [1, 2]. In high-endemic areas, mother-to-child transmission (MTCT) is the main route of HBV infection. According to guidelines for the prevention of CHB, passive-active combined immunization can reduce the rate of MTCT from 75–90% to 10%. Infants received 100 IU HB immunoglobulin (HBIG) intramuscularly and 10 μg HB vaccine within 12 h after birth, with additional HB vaccination at 1 and 6 months. However, immunoprophylaxis failure rate is 5–10% in infants born to mothers positive for HBsAg and HB e antigen (HBeAg) [3, 4].

HBIG injection in late pregnancy seems to have little effect on reducing MTCT of HBV [5–7]. At present, it is recommended that the pregnant women with high viral load take tenofovir disoproxil fumarate or telbivudine orally in the second or third trimester to reduce further the rate of MTCT, but the long-term safety of mothers and infants and hepatitis flare after postpartum discontinuation are controversial [8].

HB vaccine has 95% effectiveness in preventing HB infection and has a good safety record [1]. Many previous studies have shown that 20 μg HB vaccine can significantly improve the seroprotection in adults compare to 10 μg HB vaccine [9–11]. The current recommended dose of recombinant HB vaccine for infants in China is 10 μg [12]. In our previous study, after three doses of the HB vaccine, 1.4% of infants born to HBsAg-positive mothers did not achieve a protective level (anti-HBs ≤ 10 IU/L), and 3.7% of infants had a low response level (anti-HBs 10–99 IU/L) [13]. These infants face potential infective risk in their daily lives being in close contact with HBsAg-positive mothers. Vaccine type, low birth weight, and high maternal viral load have been identified as the most important risk factors for low immune response to HBV vaccine. In addition, host genetic background also plays an important role in determining the strength of immune response to vaccination, such as variants in human leukocyte antigen (HLA) region, mitogen-activated protein kinase eight polymorphisms [14]. Few studies have been reported about the effects of increased dose of HB vaccine on infants born to HBsAg-positive mothers. Therefore, we conducted a prospective, multicenter, large-sample cohort study to evaluate the effects of increased dose of HB vaccine (20 μg) on MTCT of HBV and immune response in infants born to HBsAg-positive mothers.

## Methods

### Study design

This was a prospective, multicenter, large-sample study. Patients were recruited from 4 hospitals in Beijing, Shijiazhuang, Taiyuan, and Tongliao, China. We evaluated and compared the effects of 20 μg HB vaccine on infants born to HBsAg-positive mothers, including immunoprophylaxis failure, immune responses to vaccine, and vaccine safety. The HBsAg-positive mothers were enrolled at 24–28 weeks’ gestation, and peripheral blood samples were collected at parturition for chemical and hematological tests. Demographic information of their infants was recorded at birth, including sex, singleton status, gestational age, birth weight, delivery mode, and 1-min APGAR scores as the baseline data. The infants were divided into two groups according to their mothers’ wishes: 10 μg (0.5 mL) recombinant HB vaccine plus HBIG (10 μg group) and 20 μg (1 mL) recombinant HB vaccine plus HBIG (20 μg group). The details of informed consent are described in Additional file 1: Appendix 1, Methods. All the vaccinations were completed at the corresponding investigational sites, and the adverse events were recorded at each follow-up at 1, 6, and 12 months. At 12 months, peripheral serum samples were taken from infants after standard immunoprophylaxis, and their HBsAg, anti-HBs, HBeAg, anti-HBe, and HB core antibody (anti-HBc) were tested. If the infant was positive for HBsAg, HBV DNA was further tested. The fetal development and infant growth were evaluated at 12 months in both HB vaccine groups. The study protocol was approved by each institutional Ethics Committee and registered at Chinese Clinical Trial Registry (ChiCTR, No. ChiCTR-PRC-09000459).

### Patients

Patient screening began from January 2009 to September 2010, and the last patient visit was on October 2011. The inclusion criteria for the mothers were as follows: HBsAg positive for > 6 months; age 18–45 years; and willing to cooperate with collection of documented information from 24 to 28 weeks’ gestation, the corresponding intervention measures, follow-up, and detection according to the informed consent. Major exclusion criteria for mothers were (1) infection with hepatitis C/D, human immunodeficiency virus, *Treponema pallidum* or *Toxoplasma gondii*; (2) treated with HBIG or antiviral therapy including interferon within 6 months before or during pregnancy; (3) alanine aminotransferase (ALT) ≥ 2× upper limit of normal (ULN) or total bilirubin (TBIL) ≥ 2 mg/dL (34.2 μmol/L), indicating cirrhosis and other liver diseases; (4) malignant tumor or definite disease in the cardiovascular, respiratory, urinary, nervous, digestive, blood, endocrine, or metabolic systems; (5) long-term use of hormones or immunosuppressive agents; and (6) taking part in other studies. The major exclusion criteria for infants were (1) prematurity (born at less than 36 weeks’ gestation), (2) birth weight < 2000 g, (3) congenital malformation, and (4) taking part in other studies.

### Immunization schedule

All infants born to HBsAg-positive mothers had the following prophylaxis schedule: the first dose of 200 IU HBIG (Chengdu Institute of Biological Products, China or Hualan Biological Engineering Inc., China) and the first dose of 10 μg (0.5 mL) or 20 μg (1 mL) recombinant HB vaccine (Hansenula yeast vaccine; Dalian Hissen Biopharm Co., China) were given intramuscularly within 2 h of birth at different sites. The second injection of the same dose of HBIG was administered at 1 month of age. The second and third doses of recombinant HB vaccines were given at 1 and 6 months of age, respectively.

### Serum biochemistry and HBV markers

All serum specimens were tested in the hospital central laboratory. The presence of HBsAg, anti-HBs, HBeAg, anti-HBe, and anti-HBc was determined using an electrical chemiluminescence immunoassay (Roche Laboratories, Mannheim, Germany) or chemiluminescent microparticle immunoassay kit (Architect i2000 analyzer; Abbott Diagnostics, Abbott Park, IL, USA). All the serum samples for HBV DNA were tested by real-time polymerase chain reaction with a range of 2–8 log_10_ IU/mL (Hunan Shengxiang Bio-engineering). ALT was measured by a fully automatic biochemical analyzer (AU5400; Olympus Optical, Tokyo, Japan). ALT > 40 IU/L was considered abnormal.

### Outcome assessment and definitions

The primary outcome was immunoprophylaxis failure, which was defined as infants who were HBsAg-positive at age 12 months [15, 16]. The secondary outcome was response status of infants to HB vaccine. All the HBsAg-negative infants (successful immunoprophylaxis) were divided into 4 groups as follows: anti-HBs < 10 IU/L was defined as non-responder, anti-HBs 10–100 IU/L was low-responder, anti-HBs 100–1000 IU/L was medium-responder, and anti-HBs ≥ 1000 IU/L was high-responder [13].

### Statistical analysis

The database was established with EpiData 3.02. Continuous variables values were expressed as the mean ± standard deviation (SD); categorical variables were expressed as percentages. The characteristics of infants who received different doses of the HB vaccine were compared by independent t-test and/or χ^2^ test or Fisher’s exact test. The maternal HBV DNA level, ALT, and TBIL in two groups were compared by the Mann-Whitney U-test. The immunoprophylaxis failure rates (MTCT of HBV) in two doses of HB vaccines were cooperated, and the planned sample size of 955 patients was estimated to provide at least 85% power to detect an absolute difference of 3% in the proportion of infants with HBV infection at 12 months on the basis of two-tailed α = 0.05 and assuming an MTCT rate of 5% in the 10 μg HB vaccine group. A multivariate logistic regression model was fitted with a stepwise method (likelihood ratio test) using significant baseline characteristics (candidate variables such as mother age, HBV DNA ≥ 5 log_10_ IU/mL, and other clinical indicators with *P* < 0.20 in Table 1) that had been prefiltered in univariate analysis to identify factors independently associated with vaccine dose grouping. The response rates of different degrees in infants between two doses of groups were compared by χ^2^ test or Fisher’s exact test. The developmental indexes of infants at 12 months between two groups were compared by independent t-test or Mann-Whitney U-test. The data were analyzed using SPSS version 17.0 (SPSS Inc., Chicago, IL, USA) or GraphPad Prism version 5.0 (GraphPad Software Inc., San Diego, CA, USA). A two-sided *P* < 0.05 was considered statistically significant.

**Table 1:** Main characteristics of HBsAg-positive mothers and their babies from two different dose of HB vaccine

Variables	10 μg (n = 478)	20 μg (n = 477)	*P* value
Maternal data			
Age (years)	27.3 ± 4.5	26.8 ± 4.6	0.076
HBeAg positive	154 (32.2%)	270 (55.0%)	< 0.001
HBV DNA (log_10_ IU/mL)	2.6 ± 2.9	3.5 ± 3.2	< 0.001
HBV DNA ≥ 5 (log_10_ IU/mL)	128	226	< 0.001
ALT (IU/L)	15.2 ± 8.6	15.6 ± 13.4	0.593
TBIL (μmol/L)	9.9 ± 6.5	9.3 ± 4.0	0.161
Infant data at birth			
Sex (male)	264	270	0.669
Gestation days	277.0 ± 6.9	278.4 ± 7.3	0.450
Birth mode (vaginal delivery)	184 (38.4%)	210 (44.0%)	0.083
Birth weight (g)	3625 ± 349.0	3508 ± 380.1	0.150
1-min APGAR	9.6 ± 0.1	9.5 ± 0.2	0.140

*Abbreviations*: *ALT* alanine aminotransferase, *HBeAg* hepatitis B e antigen, *HBsAg* hepatitis B surface antigen, *HBV* hepatitis B virus, *TBIL* total bilirubin

## Results

### Study populations

**Fig. 1:**
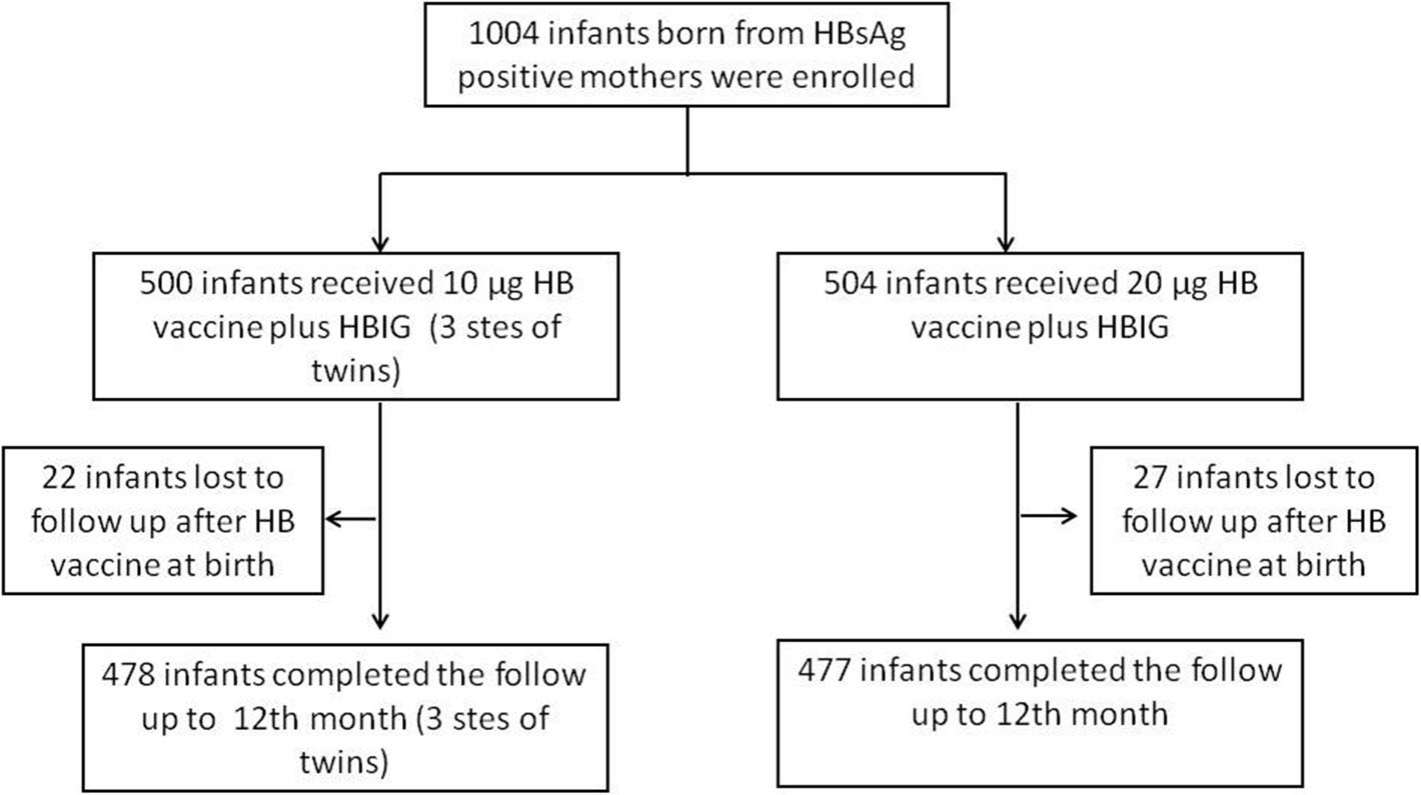
The flow chart of subject recruitment for this study. Abbreviation: HBsAg, hepatitis B surface antigen

The study was performed at 4 investigational sites in China. A total of 1004 infants born to 1001 HBsAg-positive mothers (3 mothers had twins) were enrolled, and the numbers of patients enrolled from each site were as follows: 668 in Beijing, 108 in Taiyuan, 125 in Shijiazhuang, and 100 in Tongliao. At 12 months, a total of 955 infants completed the study, there were 478 infants who completed the follow-up in the 10 μg group, and 477 infants completed the follow-up in 20 μg HB group (Fig. 1); the groupings of these infants in respective site are showed in Additional file 1: Appendix 2 Table S1. The demographic characteristics of infants at birth and their mothers with chronic HBV infection in each group are shown in Table 1.

As more HBeAg-positive mothers with or without high load of HBV DNA prefer to choose high dose of HB vaccine (20 μg) for their infants, therefore, both the HBeAg-positive rate and HBV DNA levels in mothers in the 20 μg group were higher than those in the 10 μg group (both *P* < 0.001, Table 1). Further, we used multivariable logistic analysis to screen the possible confounders (in Table 1) that lead to the differences between the high and low vaccine groups. Results showed that the high level of maternal HBV DNA ( ≥5 log_10_ IU/mL) was the independent factor of differences in baseline characteristics between two dose groups (*P* < 0.001) (OR = 0.481, 95% CI 0.362–0.639). Therefore, we did stratified analysis in the following investigation according to the maternal HBV DNA level.

### The outcomes to HB vaccines in infants born to HBsAg-positive mothers

Of the 955 infants after the standard immunization schedule, 13 infants were positive for HBsAg at 12 months, who were considered as immunoprophylaxis failure, and 942 were negative for HBsAg, who were considered as immunoprophylaxis success. The total rate of immunoprophylaxis failure was 1.4% (13/955). The characteristics of infants and their mothers between the two outcomes are shown in Additional file 1: Appendix 2 Table S2; high level of maternal HBV DNA and HBeAg-positive were the major differences between immunoprophylaxis failure and success. Among the 13 HBsAg-positive infants, 5 were in the 10 μg group and 8 were in the 20 μg group. The baseline characteristics of the infants at birth and HBV infection status at age 12 months are shown in Table 2. At 12 months, all the 13 HBsAg-positive infants were positive for HBeAg and anti-HBc.

**Table 2:** Main characteristics of HBV-infected babies in two different doses of HB vaccine

Variables	10 μg (n = 5)	20 μg (n = 8)
**At birth**		
Sex (M)	4	2
Gestation days	278.6 ± 3.5	277.5 ± 2.1
Birth mode (vaginal delivery)	2	4
Birth weight (g)	3480 ± 165.5	3300 ± 131.3
Fetal distress	1	2
1-min APGAR	9.6 ± 0.2	9.6 ± 0.18
**At 12th month**		
HBeAg +	5	5
Anti-HBc	5	8
HBV DNA detectable	3^a^	6^b^
HBV DNA (log_10_ IU/mL)	6.7 ± 0.3	6.5 ± 0.2

^a^10 μg: samples of 2 cases were unavailable

^b^20 μg: 1 case HBV DNA < 100 IU/mL, samples of 2 cases were unavailable

*Abbreviations*: *HB* hepatitis B, *Anti-HBc* antibody to hepatitis B c antigen, *HBeAg* hepatitis B e antigen, *HBV* hepatitis B virus

### The response differences to HB vaccine between low-dose and high-dose groups

Regarding the influence of high level of maternal HBV DNA, we compared the immunoprophylaxis failure rate and response status of infants between 10 μg and 20 μg groups by stratified analysis. There were 601 infants born to HBsAg-positive mothers with HBV DNA < 5 log_10_ IU/mL, 350 were in the 10 μg group and 251 were in the 20 μg group. None of the infants was positive for HBsAg in the 10 μg group (0%, 0/350), and 1 was positive in the 20 μg group (0.4%, 1/251); the immunoprophylaxis failure rates between two groups were not significantly different (*P* = 0.418, Table 3). However, the high-response rate in the 20 μg group (42.2%, 106/251) was evidently higher than that in the 10 μg group (22.9%, 80/350) (*P* < 0.001); on the contrary, the low-response rate (*P* = 0.002) and middle-response rate (*P* = 0.022) in the 20 μg group were both lower than those in the 10 μg group (Table 3). There was no difference in non-response rate between two groups (Table 3).

**Table 3:** The response differences to HB vaccine in infants born to HBsAg-positive mothers in two groups

Infant response	Maternal HBV DNA < 5 log_10_ IU/mL			Maternal HBV DNA ≥ 5 log_10_ IU/mL		
	10 μg (n = 350)	20 μg (n = 251)	*P* value	10 μg (n = 128)	20 μg (n = 226)	*P* value
Failure	0 (0%)	1 (0.4%)	0.418	5 (3.9%)	7 (3.1%)	0.922
Non-responder	11 (3.1%)	5 (2.0%)	0.387	6 (4.7%)	2 (0.9%)	0.052
Low-responder	67 (19.1%)	25 (10.0%)	0.002	17 (13.3%)	24 (10.6%)	0.452
Middle-responder	192 (54.9%)	114 (45.4%)	0.022	62 (48.4%)	124 (54.9%)	0.244
High-responder	80 (22.9%)	106 (42.2%)	< 0.001	38 (29.7%)	69 (30.5%)	0.868

*Abbreviations*: *HB* hepatitis B, *HBsAg* hepatitis B surface antigen

In the 354 infants born to mothers with high load of HBV DNA (≥ 5 log_10_ IU/mL), 128 were in the 10 μg group and 226 were in the 20 μg group. Among them, 5 infants (3.9%, 5/128) were HBsAg-positive in the 10 μg group and 7 infants (3.1%, 7/226) were HBsAg-positive in the 20 μg group. The immunoprophylaxis failure rates between two groups were not obviously different (*P* = 0.922). Interestingly, there were no significant differences in response rates of various levels between two groups, from non-response to high-response (Table 3). These were different from the infants born to mothers with low load of HBV DNA.

### High dose of HB vaccine safety for infants

Of the 955 infants who finished follow-up, the adverse events were reported in 9 infants: 4 in the 10 μg HB vaccine group (0.8%, 4/478) and 5 in the 20 μg HB vaccine group (1.0%, 5/477). Among the 9 infants, 5 had adverse injection reactions (local swelling and induration) to the first dose of vaccine (2 in the 10 μg group and 3 in the 20 μg group). Additionally, 2 cases developed fever (1 in each group), and 2 had hives (10 μg group). No severe adverse events were reported to vaccination.

We also evaluated the safety of 20 μg HB vaccine by observing the growth indexes of infants at age 12 months. There were no differences in these growth indexes between the two groups, except for body length, which was longer in the 20 μg vaccine group (*P* = 0.04) (Table 4), but still within the normal range of Chinese children’s growth and development indicators [17]. The results suggested that 20 μg HB vaccine is safe for infants.

**Table 4:** Developmental index of infants between two doses of HB vaccine at 12 months

Developmental index	10 μg (n = 478)	20 μg (n = 477)	*P* value
Weight (kg)	10.6 ± 1.5	10.6 ± 1.3	0.89
Body length (cm)	77.5 ± 3.9	78.0 ± 3.8	0.04
Head circumference (cm)	46.3 ± 1.3	46.4 ± 1.3	0.43
Abdominal fat thickness (cm)	1.5 ± 0.5	1.7 ± 2.9	0.13

*Abbreviation*: *HB* hepatitis B

## Discussion

This is a prospective, multicenter, large-sample cohort study. We compared the effects of increased dose (20 μg) and routine dose (10 μg) of HB vaccine combined with HBIG on infants born to HBsAg-positive mothers. Our results revealed that high dose of HB vaccine did not reduce MTCT of HBV, but could decrease low-response rate, middle-response rate, and increase high-response rate for those infants born to mothers with low viral load (HBV DNA < 5 log_10_ IU/mL). However, for those infants born to mothers with high viral load (HBV DNA ≥ 5 log_10_ IU/mL), the above response advantages of 20 μg HB vaccine were nearly not obvious.

Maternal HBV infection status, such as HBeAg positivity, HBV DNA, and intrauterine infection, is thought to be important for HBV MTCT [18–20]. Considering the importance of maternal virological factor, we performed stratified analysis to our findings according to the maternal HBV load. Data in each group showed that 20 μg HB vaccine did not significantly reduce the MTCT of HBV, whatever maternal HBV load was high or low. The reasons for immunoprophylaxis failure are not completely clear now. It is reported HBV breach of the placental barrier largely occurs in late pregnancy because of the thinner trophocyte layer, which forms the chorionic vascular membrane that facilitates HBV passage through the thinner placental barrier [21]. Therefore, administration of nucleoside analogs during late pregnancy, such as tenofovir dipivoxil fumarate and telbivudine, is beneficial for HBsAg-positive mothers with a high viral load and could effectively reduce the intrauterine HBV infection and increase the protection of vaccine and HBIG for infants [22–24].

Although 20 μg HB vaccine did not reduce MTCT of HBV, it did influence the immune response of infants, compared with 10 μg HB vaccine. For example, for the infants born to mothers with low level of HBV DNA (< 5 log_10_ IU/mL), 20 μg HB vaccine significantly increased the high-response rate and reduced the low-response rate. A related investigation on 1192 infants born to HBsAg-positive mothers reported that 20 μg HB vaccination reduced the risk of low responsiveness in infants with HLA-II risk genotype of HBsAg-positive mothers [25]. Some investigations on healthy individuals also demonstrated that 20 μg HB vaccine could increase the anti-HBs level compared with 10 μg HB vaccine [10, 11]. Therefore, some researchers think that for the immune non-responders and low-responders, more inoculations, a higher concentration of HB vaccine to increase the immunogenicity is reasonable [3], especially for those born to mothers whose HBV DNA are < 5 log_10_ IU/mL.

However, in those infants born to mothers with high viral loads (≥ 5 log_10_ IU/mL), 20 μg HB vaccine did not show the response advantages like those happened in infants born to mothers with low viral loads. Our results suggest that maternal HBV DNA levels might be related to the responses of their infants to HB vaccine, but the mechanism is still unknown. Lazizi et al. and Badur et al. demonstrated the relationship between unresponsiveness to HB vaccine in newborns and HBV DNA from maternal peripheral blood mononuclear cells [26, 27]. Some researchers have reported that transfer of maternal cells to newborn circulation participates in the immune response in an antigen-specific manner [28, 29]. Zhang et al. found that high maternal titer of anti-HBs can transplacentally impair immune response of infants towards HB vaccine [30]. Whether a similar mechanism can explain our results, it needs further research. In light of these findings, reducing maternal viral load in late pregnancy and increasing HB vaccine dose of infants might be advantageous to produce more effective immune response to HB vaccine for infants.

There were limitations in our study. We did not further test HBV DNA levels in HBsAg-negative infants at 12 months, although occult HBV infection has been reported at a low frequency in HB-vaccinated children, especially in those with absent or low anti-HBs levels [31, 32]. This factor might influence the accuracy of immunoprophylaxis failure and non-response rates of the HB vaccine. Additionally, the long-time prevention of 20 μg HB vaccine on these infants needed further follow-up and investigation.

## Conclusion

In conclusion, increasing dose of HB vaccine (20 μg) did not further reduce the MTCT of HBV, but was helpful to enhance more effective immune response for infants born to mothers with low load of HBV DNA, by increasing high-response rate and decreasing low-response rate and middle-response rate. Our study is expected to provide clinical basis for further improving the strategy of enhancing protection of HB vaccine for infants in the perinatal period.

## Supplementary Information

**Additional file 1: Appendix 1**. Methods. The main contents of informed consents; Statistics used in Table S2. **Appendix 2**. **Table S1**. The infants who completed the final follow-up at their respective investigational sites. **Table S2**. The comparison of characteristics of the failure infants and successful infants and their mothers.

## Data Availability

In accordance with the current national law and consensus of researchers in this study, the data used in this study is only available for the researchers participating in this project. Thus, we are not allowed to distribute or make publicly available the data to other parties.

## Abbreviations

ALT: Alanine aminotransferase
anti-HBc: Hepatitis B core antibody
anti-HBs: Hepatitis B surface antibody
HB: Hepatitis B
HBeAg: HBV e antigen
HBIG: Hepatitis B immunoglobulin
HBsAg: HBV surface antigen
HBV: Hepatitis B virus
HIV: Human immunodeficiency virus
MTCT: Mother-to-child transmission
OR: Odds ratios
SD: Standard deviation
TBIL: Total bilirubin
