# Supplementary material for: The effects of increased dose of hepatitis B vaccine on mother-to-child transmission and immune response for infants born to mothers with chronic hepatitis B infection: a prospective, multicenter, large-sample cohort study
Source: BMC Med. 2021 Jul 13;19:148. doi: 10.1186/s12916-021-02025-1 (PMC8276424; doi:10.1186/s12916-021-02025-1)
Supplement: Supplementary file 1 — Additional file 1: Appendix 1. Methods. The main contents of informed consents; Statistics used in Table S2. Appendix 2. Table S1. The infants who completed the final follow-up at their respective investigational sites. Table S2. The comparison of characteristics of the failure infants and successful infants and their mothers. [file 12916_2021_2025_MOESM1_ESM.docx]

**Additional file 1**

Appendix 1. Methods. The main contents of informed consents

Statistics (Table S2)

Appendix 2. Table S1. The infants who completed the final follow-up at their respective investigational sites

Table S2. The comparison of characteristics of the failure infants and successful infants and their mothers

**Appendix 1.**

**Methods**

**The main contents of informed consents**

This study was conducted in accordance with the ethical principles that had their origin in the Declaration of Helsinki and adhere to International Conference on Harmonization Good Clinical Practice (ICH GCP), and applicable regulatory requirements. Approval from the Independent Ethics Committee was obtained before starting the study.

First of all, all the mothers who participated in this study were given adequate explanations of the research hypothesis, aims, methods (study design, number of subjects per group, study population, inclusion criteria, exclusion criteria, visit schedule and assessments and other information related to the study), the potential hazards of the study (high vs low vaccine groups).

After counseling the possible risks and benefits of high and low vaccine, the mothers themselves decided whether or not to undergo high vaccine for their infants. According to mothers’ decision, the infants were divided into two groups: 10 μg recombinant HB vaccine plus HBIG (low vaccine group, control group) and 20 μg recombinant HB vaccine plus HBIG (high vaccine group, experimental group).

At the same time, it must also be explained to the mothers that they are not obligated to enroll in the study and may withdraw consent at any time for any reason.

All mothers provided their written informed consent.

The study was registered at ClinicalTrials.gov (ChiCTR-PRC-09000459).

**Statistics (Table S2)**

The characteristics of infants and their mothers between immunoprophylaxis failure and success were compared by independent t-test, or Mann-Whitney U-test and/or χ^2^ test or Fisher’s exact test.

**Appendix 2.**

Table S1. **The infants who completed the final follow-up at their respective investigational sites**

| Investigational Site | 10 μg  (n = 478) | 20 μg  (n = 477) | Total  (n = 955) |
| --- | --- | --- | --- |
| Beijing | 301 | 324 | 625 |
| Taiyuan | 52 | 56 | 108 |
| Shijiazhuang | 63 | 61 | 124 |
| Tongliao | 62 | 36 | 98 |

Table S2. **The comparison of characteristics of the failure infants and successful infants and their mothers**

| Variables | Failure  (n = 13) | Success  (n = 942) | *P* value |
| --- | --- | --- | --- |
| Maternal data |  |  |  |
| Age | 26.2 ± 4.5 | 27.5 ± 4.2 | 0.333 |
| HBeAg + | 12 (92.3%) | 464 (49.3%) | < 0.001 |
| HBV DNA, log_10_ IU/ml | 5.8 ± 0.7 | 4.5 ± 1.4 | < 0.010 |
| ALT levels (IU/L) | 17.2 ± 6.9 | 16.8 ± 14.2 | 0.837 |
| TBIL (μmol/L) | 10.4 ± 4.6 | 10.6 ± 4.7 | 0.978 |
| Infant data |  |  |  |
| Sex (male) | 5 | 550 | 0.149 |
| Gestation days | 277.9 ± 6.4 | 276.1 ± 8.5 | 0.459 |
| Birth mode  (Vaginal delivery) | 6 | 381 | 0.684 |
| Birth weight (g) | 3369 ± 366.6 | 3531 ± 435.1 | 0.217 |
| 1 min APGAR | 9.6 ± 0.5 | 9.4 ± 0.6 | 0.301 |

Abbreviation: ALT, alanine aminotransferase; HBeAg, hepatitis B e antigen; HBV, hepatitis B virus
